# Aurora kinase a promotes the progression of papillary thyroid carcinoma by activating the mTORC2-AKT signalling pathway

**DOI:** 10.1186/s13578-022-00934-z

**Published:** 2022-12-05

**Authors:** Zewei Zhao, Huijuan Wang, Ning Kang, Zhongyu Wang, Xiukun Hou, Linfei Hu, Shuo Qie, Jianping Guo, Songfeng Wei, Xianhui Ruan, Xiangqian Zheng

**Affiliations:** 1 Department of Thyroid and Neck Cancer, Tianjin Medical University Cancer Institute and Hospital, National Clinical Research Center for Cancer, Key Laboratory of Cancer Prevention and Therapy, Tianjin’s Clinical Research Center for Cancer, Tianjin, 300060 China; 2 Department of Pathology, Tianjin Medical University Cancer Institute and Hospital, National Clinical Research Center for Cancer, Key Laboratory of Cancer Prevention and Therapy, Tianjin’s Clinical Research Center for Cancer, Tianjin, 300060 China; 3 Institute of Precision Medicine, The First Affiliated Hospital, Sun Yat-Sen University, Guangzhou, 510275 Guangdong China

**Keywords:** Papillary thyroid carcinoma, AURKA, SIN1, AKT, CUL4B

## Abstract

**Background:**

Treatment failure is the main cause of death from papillary thyroid carcinoma (PTC). It is urgent to look for new intervention targets and to develop new therapies for treating PTC. Aurora-A kinase (AURKA) functionally regulates cell mitosis and is closely related to the occurrence and development of a variety of tumours. However, the expression and potential functions of AURKA in PTC remain largely elusive.

**Results:**

Clinicopathologically, AURKA is highly expressed in PTC tissues compared to normal tissues and is correlated with lymph node metastasis, TNM stage and patient prognosis. Biologically, AURKA functions as an oncoprotein to promote the proliferation and migration of PTC cells. Mechanistically, AURKA directly binds to SIN1 and compromises CUL4B-based E3 ligase-mediated ubiquitination and subsequent degradation of SIN1, leading to hyperactivation of the mTORC2-AKT pathway in PTC cells.

**Conclusions:**

We found that AURKA plays critical roles in regulating the progression of PTC by activating the mTORC2-AKT pathway, highlighting the potential of targeting AURKA to treat PTC.

**Supplementary Information:**

The online version contains supplementary material available at 10.1186/s13578-022-00934-z.

## Background

Thyroid carcinoma (TC) is the most common endocrine system malignancy. In 2020, cancer data from 185 countries showed that thyroid cancer accounted for 3.0% of new tumours, while in China, thyroid cancer accounted for approximately 4.8% of all tumours [1, 2]. Papillary thyroid carcinoma (PTC) accounts for 90–97% of thyroid cancers, and the vast majority of PTC patients have a good prognosis. However, 20–30% of patients experience recurrence, and 5–10% of them develop refractory thyroid cancer. Therefore, more accurate risk stratification of PTC and the search for new, individualized intervention targets have become the top priorities for improving PTC diagnosis and treatment [3].

AURKA is a mitotic serine/threonine-protein kinase that plays different roles in the proliferation, migration, stemness maintenance and other biological behaviours of tumour cells [4]. Overexpression of AURKA has been noted in various tumour tissues, such as those of head and neck tumours, breast cancer, non-small cell lung cancer, ovarian cancer, oesophageal cancer, gastric cancer, colon cancer, and anaplastic thyroid cancer, and is correlated with poor prognosis and tumour progression [5–8]. AURKA is involved in the regulation of multiple signalling pathways, enabling tumour cells to escape apoptosis and proliferate massively. By activating the IKK/NF-κB pathway and the PI3K/Akt/GSK3 signalling cascade, AURKA improves cell anti-apoptotic ability and resistance to chemical drugs [9–12]. For tumour cell migration, AURKA induces the expression of SLUG, FNB1, and MMP and activates signalling pathways that promote tumour cell migration, including AKT, MAPK, cofilin-F-actin, SRC, and MCAK, to promote tumour cell migration [13–17]. A previous study reported that the AURKA inhibitor MLN8237 inhibits thyroid cancer growth by promoting the proteasomal degradation of c-Myc [18]. Additionally, studies have shown that AURKA is overexpressed in PTC, which means that AURKA may play a relevant role and may represent a therapeutic target for PTC. However, the specific function and mechanism of AURKA in PTC have not been described before.

Stress-activated protein kinase (SAPK)-interacting protein 1 (SIN1) is a key subunit of mTORC2 and a critical regulator of the AKT pathway that plays important roles in various pathological processes of cancers. Phosphorylated SIN1 dissociates SIN1 from mTORC2 and suppresses mTORC2 kinase activity, consequently inhibiting downstream AKT signalling to suppress tumorigenesis [19]. Moreover, PtdIns(3,4,5)P3 interacts with the PH domain of SIN1 to trigger the activation of mTORC2 [20]. In PTC, phospho-mTOR activation leads to the activation of the mTORC2 complex and phosphorylation of AKT at Ser473, which are involved in distant metastasis and therapy resistance [21]. However, the functional role of SIN1 in PTC has not been well characterized.

In this study, we found that AURKA is highly expressed in PTC and can affect the progression of PTC in vitro and in vivo. AURKA affects SIN1 stability by competing with CUL4B for binding to SIN1, an important subunit of mTORC2, thereby mediating the activation of the PI3K/mTORC2/AKT signalling pathway, which is involved in the occurrence and development of PTC.

## Results

### AURKA is highly expressed in PTC

**Fig. 1:**
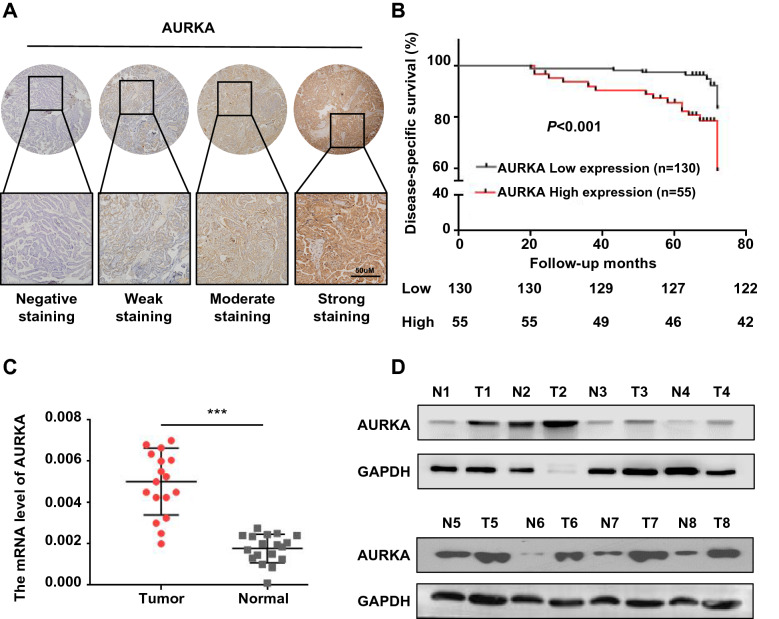
AURKA is highly expressed in thyroid cancer and correlated with a poor prognosis of PTC. **A** Representative IHC analysis of AURKA expression in different thyroid cancer patient specimens. The scale bar is 50 μm. **B** The disease-specific survival rate of the high AURKA expression group was compared with that of the low AURKA expression group (*P* < 0.001). **C** Quantitative real-time PCR to measure the mRNA level of AURKA in normal and cancer tissues. **D** The protein expression levels of AURKA were examined by western blotting in normal and cancer tissues

To detect the relationship between the expression of AURKA and the clinicopathological features of the patients, we performed immunohistochemical staining on tissue microarrays including 185 PTC tissue samples. The results indicated that AURKA was expressed in both the nucleus and cytoplasm (Fig. 1A). Chi-square analysis suggested that high AURKA expression was positively correlated with advanced T staging, lymph node metastasis and TNM staging (Additional file 2: Table S1). Moreover, survival analysis showed that patients with high expression of AURKA had poor disease-specific survival. The median follow-up of these 185 patients was 65 months (range from 9 to 72 months). Among these patients, 30 experienced recurrence or persistent disease, and 21 died from cancer-related causes. In the low AURKA expression group, 122 (93.8%) patients survived, and 8 patients (6.2%) died; In the high AURKA expression group, 42 (76.4%) patients survived, and 13 patients (23.6%) died (Fig. 1B). To further detect the expression of AURKA in PTC, we used RT–qPCR to detect the mRNA level of AURKA in fresh PTC tissues and matched normal tissues from 18 patients. The mRNA levels of AURKA in PTC tissue were significantly higher than those in normal thyroid tissue (Fig. 1C). Furthermore, eight pairs of thyroid cancer and adjacent normal tissues were selected to detect the expression of AURKA by western blotting. Thyroid cancer tissues showed higher AURKA expression than adjacent normal tissues (Fig. 1D). Similarly, the mRNA and protein levels of AURKA in normal thyroid epithelial cells were lower than those in PTC cell lines (Additional file 1: Fig. S1A&B). These findings suggest that elevated AURKA expression in PTC is correlated with aggressive clinicopathological features and is an independent poor prognostic factor in PTC patients.

### AURKA acts as an oncoprotein in PTC

**Fig. 2:**
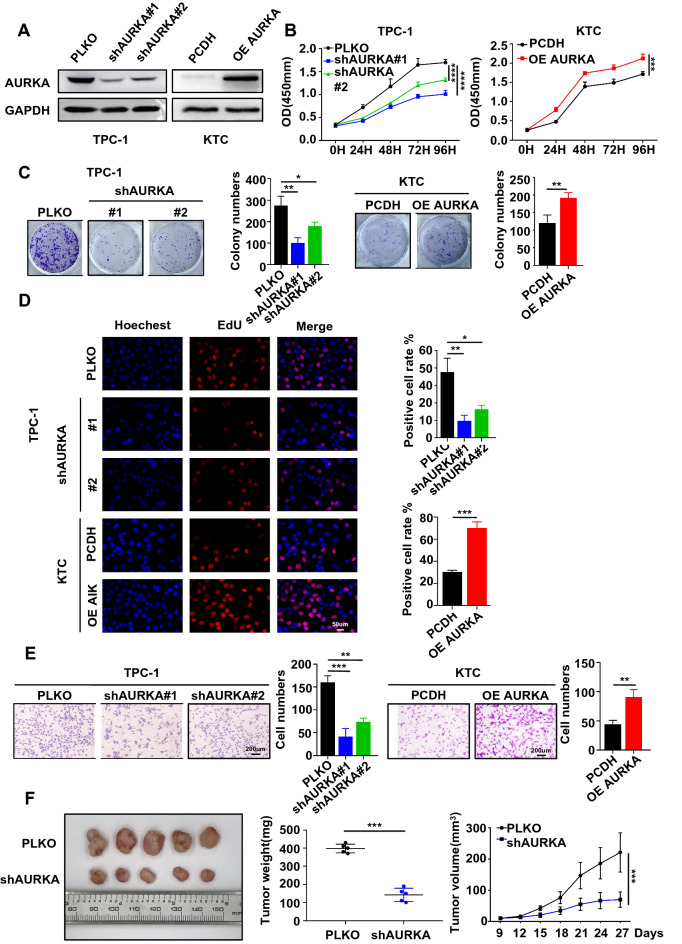
AURKA acts as an oncoprotein in PTC. **A** The protein level of AURKA was detected in AURKA knockdown TPC-1 cells and AURKA-overexpressing KTC cells by western blotting. **B** The viability of shAURKA-transfected TPC-1 cells or AURKA-overexpressing plasmid-transfected KTC cells compared with control cells was detected using the CCK-8 assay. **C** The shAURKA-transfected TPC-1 cells and AURKA-overexpression plasmid-transfected KTC cells were cultured for 10 days prior to crystal violet staining. **D** The ratio of EdU-labelled cells to total cells was observed in AURKA knockdown TPC-1 cells and AURKA-overexpressing KTC cells compared with the control group (original magnification × 20). **E** Transwell migration assays were used to detect the migration ability of AURKA knockdown TPC-1 cells and AURKA-overexpressing KTC cells compared with control cells. **F** Representative images of subcutaneous xenografts in nude mice derived from PLKO-transfected TPC-1 cells and shAURKA-transfected TPC-1 cells. n = 5 mice per group. Data are shown as the mean ± SD of three replicates (*P < 0.05, **P < 0.01, ***P < 0.001)

To assess the function of AURKA in thyroid cancer in vitro, we constructed cell lines with stable knockdown and overexpression of AURKA (Fig. 2A) and observed that knockdown of AURKA in PTC cells significantly decreased the viability of TPC-1 cells and overexpression of AURKA increased the viability of KTC cells (Fig. 2B). The ability of AURKA to increase the proliferative capacity of KTC cells and restrict the proliferative capacity of TPC-1 cells was further validated by using a colony formation assay (Fig. 2C) and an EdU staining assay (Fig. 2D). Moreover, a transwell experiment was employed and showed that knockdown of AURKA inhibited the migration of TPC-1 cells and overexpression of AURKA promoted the migration of KTC cells (Fig. 2E).

To further check whether increasing AURKA expression by activating its endogenous promoter can phenocopy the effects of ectopic AURKA expression, the CRISPRa technique was employed in K1 cells with low AURKA expression (Additional file 1: Fig. S2A). Accordingly, AURKA expression was successfully induced in K1 cells (Additional file 1: Fig. S2B). CCK-8, colony formation, EdU staining and transwell migration assays showed that overexpression of AURKA promoted the proliferation and migration of K1 cells (Additional file 1: Fig. S2C–F). We also successfully knocked down AURKA stably in IHH4 cells (Additional file 1: Fig. S2A) and observed that knockdown of AURKA prevented proliferation, colony formation and migration (Additional file 1: Fig. S2C–F).

To further explore the physiological role of AURKA in vivo, we generated xenografts with TPC-1 cells with stable knockdown of AURKA. Compared with those of control group tumours, knocking down AURKA markedly decreased tumour volumes and weights (Fig. 2F). Additionally, IHC staining showed fewer Ki-67-positive cells in AURKA-depleted tumour tissues than in the control group (Additional file 1: Fig. S2G). This finding is consistent with our previous finding that AURKA knockdown inhibits PTC cell proliferation in vitro. In brief, we found that AURKA plays important roles in PTC growth both in vitro and in vivo.

### AURKA activates AKT kinase in PTC

**Fig. 3:**
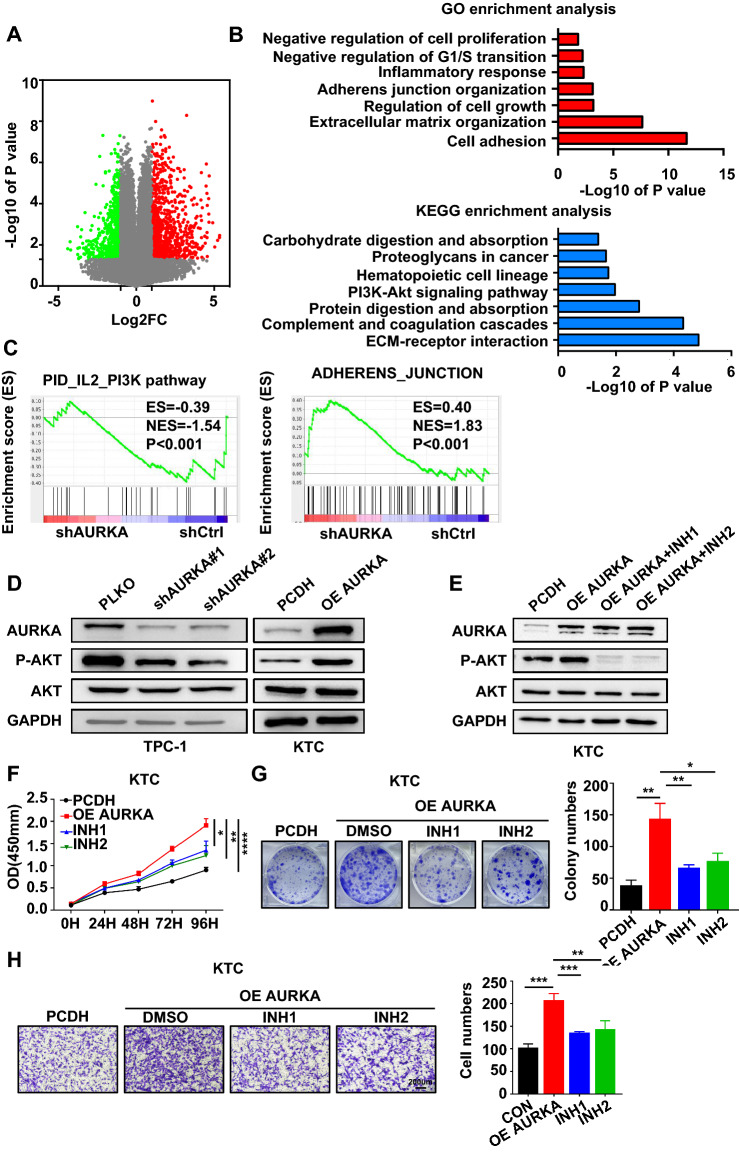
AURKA activates AKT kinase in PTC. **A** Volcano plot of the RNA-seq analysis results for TPC-1 cells after transfection with shAURKA. **B** Representative KEGG pathway analysis and GO term analysis of upregulated and downregulated genes after transfection with shAURKA. **C** GSEA showed that genes differentially expressed after AURKA knockdown were significantly enriched in the gene sets related to the P13K/AKT signalling pathway and adhesion. **D** Western blotting validation of PI3K/AKT signalling pathway component expression in AURKA knockdown TPC-1 cells and AURKA-overexpressing KTC cells. **E** AURKA overexpression plasmid-transfected KTC cells were maintained in medium containing an AKT inhibitor for 48 h, and then the levels of P-AKT were detected by western blotting. **F** KTC cells overexpressing AURKA were maintained in medium containing an AKT inhibitor for 48 h, and cell viability was determined using a CCK-8 kit. **G&H** AURKA-overexpressing KTC cells were maintained in medium containing an AKT inhibitor for 48 h, followed by clonogenic and transwell migration assays. Data are shown as the mean ± SD of three replicates (*P < 0.05, **P < 0.01, ***P < 0.001)

The potential mechanism of AURKA in PTC development remains to be elucidated. Therefore, we performed a high-throughput transcriptional study (RNA-seq) with AURKA knockdown TPC-1 cells and control cells (Fig. 3A). RNA-seq identified 1695 genes whose expression was altered after AURKA was knocked down. We then functionally classified 814 upregulated and 881 downregulated genes based on Gene Ontology (GO) terms (in the biological process category). These genes were involved in a variety of biological processes, especially signalling pathways such as cell growth and cell adhesion (Fig. 3B).

Interestingly, both Kyoto Encyclopedia of Genes and Genomes (KEGG) and gene set enrichment analysis (GSEA) analyses indicated that the PI3K-AKT signalling pathway enrichment was significantly decreased upon AURKA knockdown (Fig. 3B&C). In addition, we also verified by western blotting that the expression of phosphorylated AKT (P-AKT) decreased when AURKA was downregulated, while the overexpression of AURKA increased P-AKT levels (Fig. 3D and Additional file 1: Fig. S3A). For mouse subcutaneous tumours, P-AKT was also significantly downregulated in the AURKA low-expressing group compared to the control (Additional file 1: Fig. S3B). Furthermore, we tested whether AURKA regulates AKT phosphorylation via mTOR, a pivotal upstream kinase complex that functions to control AKT phosphorylation and downstream signalling. Ridaforolimus and tacrolimus, mTOR inhibitors, were applied in stable cell lines overexpressing AURKA and strongly repressed AURKA-induced P-AKT levels (Fig. 3E), indicating that AURKA may activate the AKT signalling pathway through mTOR. To verify whether AURKA exerted its function in PTC by activating Akt kinase, we found that the mTOR inhibitor can significantly reverse the AURKA-induced PCT malignancy phenotype (Fig. 3F–H). Thus, AURKA enhances AKT phosphorylation through its upstream kinase mTOR, promoting the proliferation and migration of PTC cells.

### AURKA interacts with and stabilizes SIN1

**Fig. 4:**
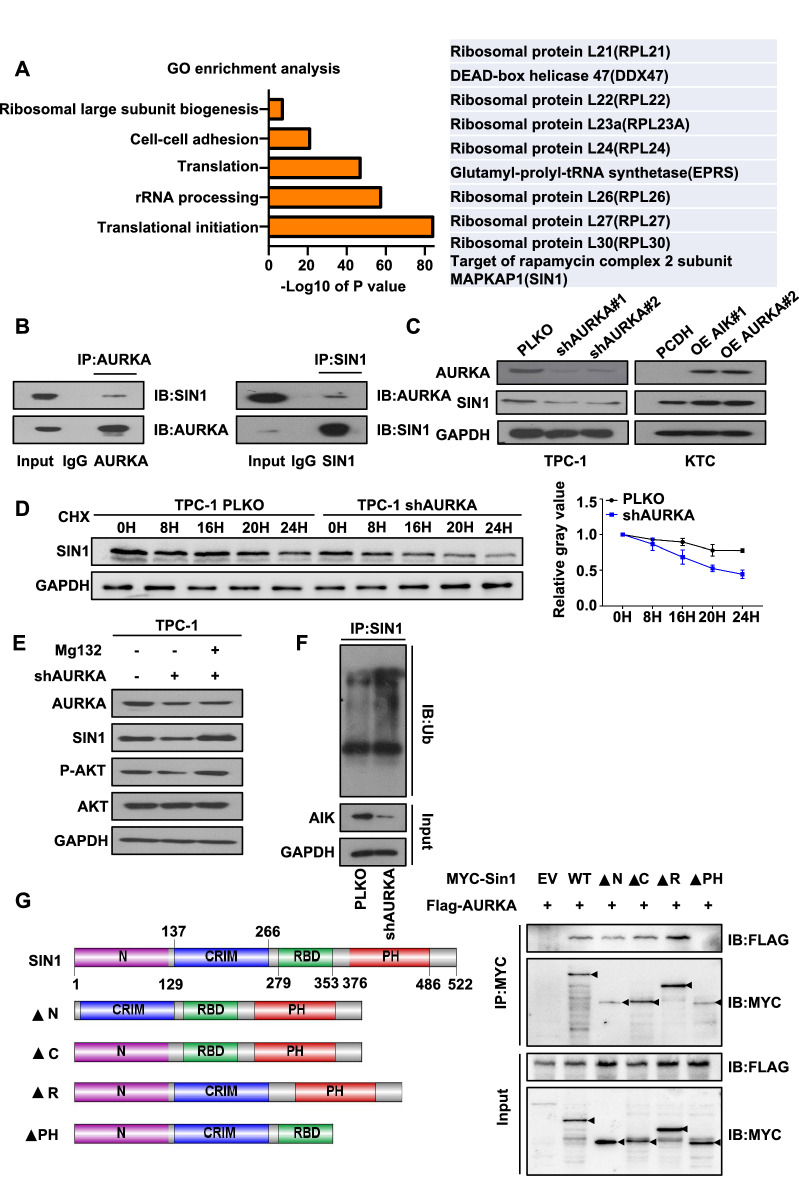
AURKA interacts with and stabilizes SIN1. **A** IP assays coupled with mass spectrometry analysis in OE AURKA-Flag-transfected KTC cells. Representative GO term analysis of upregulated and downregulated genes after transfection with shAURKA. Representative GO term analysis of proteins bound to AURKA after IP experiments. **B** The interaction of AURKA and SIN1 was detected at endogenous levels in TPC-1 cells. Mg132 was added 6 h prior to cell harvest for immunoprecipitation. Western blotting was performed with the indicated antibodies as shown. **C** The protein expression levels of SIN1 were examined by western blotting in shAURKA-transfected TPC-1 cells and AURKA-overexpression plasmid-transfected KTC cells. **D** The protein expression of SIN1 in TPC-1 cells treated with 200 μM CHX for different durations was measured by western blotting analysis. **E** The protein expression levels of SIN1 and P-AKT were examined by western blotting in shAURKA-transfected TPC-1 cells, and Mg132 (20 μM) was added 6 h prior to cell harvesting. **F** AURKA knockdown increased the endogenous ubiquitination of SIN1. TPC-1 cells with or without AURKA knockdown were treated with Mg132 (20 μM) for 6 h. Then, the total and ubiquitinated SIN1 levels were analysed by western blotting. **G** Various SIN1 truncations were generated, and their interaction with AURKA was examined by transfection-IP experiments in 293 T cells. Mg132 (20 μM) was added 6 h prior to harvest

To further explore the mechanism of AURKA function in a PTC cell line, we used the KTC cell line stably overexpressing Flag-AURKA, and through an immunoprecipitation experiment with a Flag tag, we identified the proteins that may be bound to AURKA. Interestingly, we discovered SIN1, an important subunit of mTORC2, interacts with AURKA (Fig. 4A). Consistent with this finding, we also found that SIN1 and AURKA colocalized in PTC cells (Additional file 1: Fig. S3C). Moreover, the interaction between AURKA and SIN1 at endogenous levels was also observed in TPC-1 cells (Fig. 4B). According to western blot analysis, SIN1 expression levels were correlated with AURKA expression levels, as AURKA overexpression led to elevated expression of the SIN1 protein, whereas AURKA depletion was associated with low SIN1 protein levels (Fig. 4C). We confirmed these results in IHH4 cells and K1 cells (Additional file 1: Fig. S3D). For mouse subcutaneous tumours, SIN1 was also downregulated in the AURKA low-expressing group compared to the control (Additional file 1: Fig. S3E). Moreover, upon knockdown or overexpression of AURKA, we found minor changes in SIN1 mRNA levels (Additional file 1: Fig. S3F), suggesting that AURKA modulates SIN1 expression, probably at the posttranscriptional level.

To demonstrate that AURKA regulates the expression of SIN1 by affecting its protein stability, we performed a cycloheximide pulse-chase (CHX) experiment and found that depletion of AURKA accelerated SIN1 protein decay (Fig. 4D). Additionally, SIN1 showed lower protein levels in the shAURKA group compared to the control group, and the effect of shAURKA could be rescued by Mg132 (Fig. 4E). Consistent with previous results, SIN1 showed higher levels of ubiquitination in the shAURKA group compared to the control group (Fig. 4F). Next, we constructed four truncated plasmids, N, CRIM, RBD, and PH, according to the functional domain of SIN1. Notably, the PH domain of SIN1, but not N, CRIM or RBD, interacted with AURKA (Fig. 4G). These results indicated that AURKA promotes the mTORC2/AKT signalling pathway by repressing SIN1 ubiquitination and subsequent degradation.

### AURKA stabilizes SIN1 by blocking its interaction with CUL4B

**Fig. 5:**
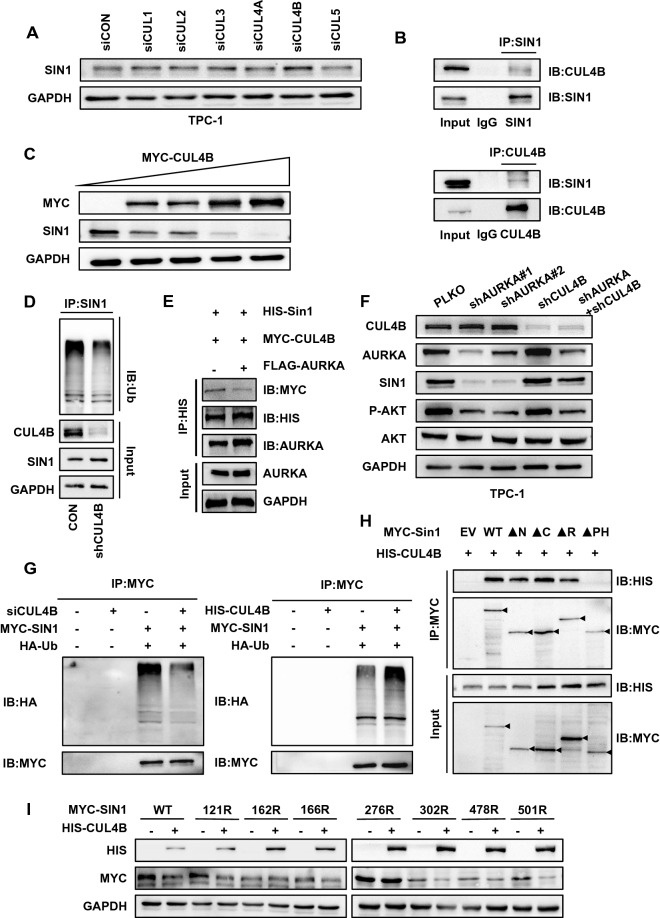
AURKA stabilizes SIN1 by blocking its interaction with CUL4B. **A** TPC-1 cells were transfected with siRNA targeting various Cullins, and the expression level of SIN1 protein was analysed by western blotting. **B** The interaction of CUL4B and SIN1 was detected at the endogenous level in TPC-1 cells. Mg132 (20 μM) was added 6 h prior to cell harvest for immunoprecipitation. **C** 293 T cells were transfected with the indicated plasmids and analysed by western blotting. Increasing CUL4B expression reduced the expression of SIN1. Mg132 (20 μM) was added 6 h prior to harvest. **D** CUL4B knockdown reduced the endogenous ubiquitination of SIN1. TPC-1 cells with or without CUL4B knockdown were treated with Mg132 (20 μM) for 6 h. Then, the total and ubiquitinated SIN1 levels were analysed by western blotting. **E** 293 T cells were transfected with the indicated plasmids, and His-IP was performed and analysed by western blotting. The expression of AURKA reduces the binding between SIN1 and CUL4B. Mg132 (20 μM) was added 6 h prior to harvest. **F** Western blotting validation of the P13K/AKT signalling pathway and the expression of SIN1 in shAURKA-transfected TPC-1 cells, shAURKA-transfected TPC-1 cells and shAURKA/shCUL4B-transfected TPC-1 cells. **G** 293 T cells were transfected with the indicated plasmids and siRNA, and MYC-IP was performed and analysed by western blotting. Mg132 (20 μM) was added 6 h prior to harvest. **H** Various SIN1 truncations were generated, and their interaction with CUL4B was examined by transfection-IP experiments in 293 T cells. Mg132 (20 μM) was added 6 h prior to harvest. **I** Various SIN1 mutants were cotransfected with CUL4B constructs to examine degradation by SIN1. Mg132 (20 μM) was added 6 h prior to harvest

Cullin-RING ubiquitin-protein ligases (CRLs) make up the largest ubiquitin E3 ligase family, and we hypothesized that CRL family E3 ligases contribute to the degradation of SIN1. Then, we knocked down Cullin proteins (CUL), which serve as scaffolds for CRL family E3 ligase members, by siRNA. Interestingly, depletion of CUL4B, but not other Cullin proteins, including CUL1, CUL2, CUL3, CUL4A, and CUL5, considerably increased SIN1 expression in TPC-1 cells (Fig. 5A & Additional file 1: Fig. S4A). Taken together, these results demonstrated that SIN1 protein was possibly regulated by the CUL4B complex. Consistent with this hypothesis, the interaction between SIN1 and CUL4B was observed at both ectopic and endogenous levels (Fig. 5B). Consistently, cycloheximide pulse-chase experiments showed that knockdown of CUL4B prolonged the half-life of SIN1 protein (Additional file 1: Fig. S4B). In contrast, ectopic expression of CUL4B decreased SIN1 levels (Fig. 5C). Ubiquitination assays further demonstrated that stable knockdown of CUL4B markedly reduced the ubiquitination level of SIN1 in the TPC-1 cell line (Fig. 5D).

More importantly, ectopically expressed AURKA significantly reduced the interaction of CUL4B and SIN1 (Fig. 5E), indicating that AURKA may compete with CUL4B in binding SIN1 and prevent SIN1 degradation. Consistent with the data above, knockdown of AURKA attenuated both SIN1 and P-AKT protein levels, and knockdown of CUL4B resulted in the upregulation of SIN1 and P-AKT. Conversely, SIN1 protein levels and P-AKT levels were restored in TPC-1 cell lines with stable knockdown of AURKA and CUL4B (Fig. 5F). We also observed that exogenous overexpression and knockdown of CUL4B resulted in increased and decreased ubiquitination of SIN1, respectively (Fig. 5G and Additional file 1: Fig. S4C). Surprisingly, CUL4B also bound to the PH domain of SIN1, and the region interacted with AURKA. Taken together, these results show that there is competition between AURKA and CUL4B for binding to SIN1 (Fig. 5H). To determine which site is targeted by CUL4B for ubiquitination, we generated various deletion mutants based on the SIN1 ubiquitination sites reported by PhosphoSitePlus (https://www.phosphosite.org/) and then tested their relative ubiquitination status in CUL4B-overexpressing cells. Among these seven identified major ubiquitination sites, K162 and K276 likely mediate CUL4B-dependent SIN1 ubiquitination and degradation, as their mutations more effectively blocked CUL4B-mediated downregulation of SIN1 (Fig. 5I). In conclusion, these results reveal that AURKA can compete with CUL4B for binding with SIN1 to prevent ubiquitination and subsequent degradation, thereby promoting the mTORC2/AKT signalling pathway.

### SIN1 mediates AURKA functions in regulating PTC progression

**Fig. 6:**
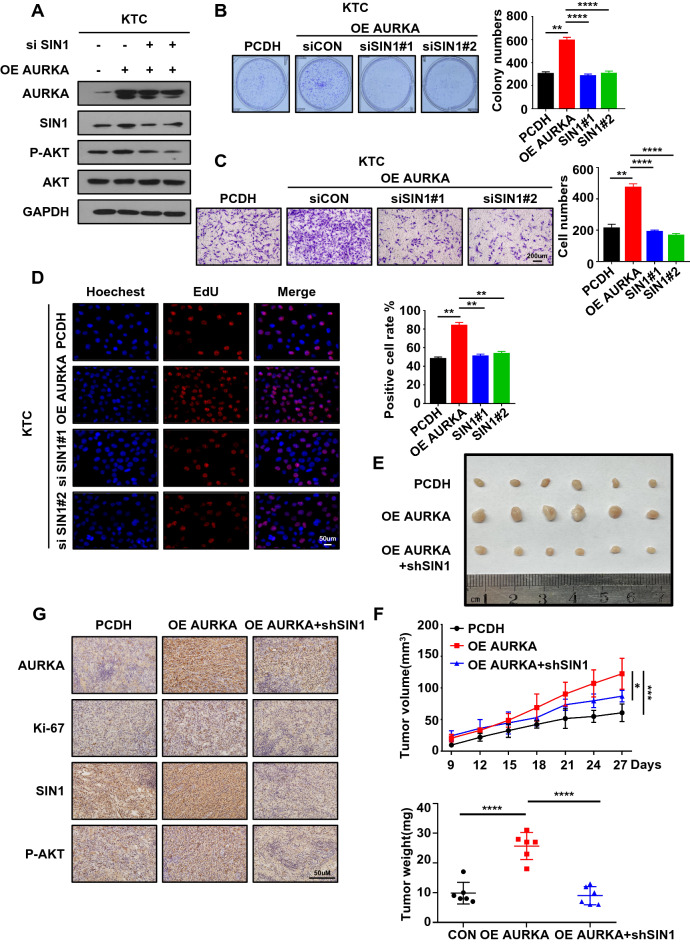
SIN1 mediates AURKA functions in regulating PTC progression. **A** The protein expression levels of SIN1 and P-AKT were examined by western blotting in OE AURKA-transfected KTC cells and siSIN1/OE AURKA-transfected KTC cells. Mg132 (20 μM) was added 6 h prior to cell harvest for WESTERN BLOT. **B** KTC cells were stably transduced with AURKA or siSIN1 were cultured for 14 days prior to crystal violet staining. **C** Transwell migration assays were carried out using KTC cells stably overexpressing AURKA and siSIN1-transfected KTC cells or the corresponding negative control (NC) cells. **D** The ratio of EdU-labelled cells was observed in AURKA-overexpressing KTC cells and siSIN1-transfected KTC cells compared with the control group. **E**, **F** Representative images of subcutaneous xenografts in nude mice derived from OE AURKA-transfected KTC cells or PCDH-transfected KTC cells or shSIN1/OE AURKA-transfected KTC cells. **G** The expression levels of AURKA, Ki67, P-AKT and SIN1 in xenografts of each group were assessed by immunohistochemical staining. The scale bar is 50 μm. Data are shown as the mean ± SD of three replicates (*P < 0.05, **P < 0.01, ***P < 0.001)

To gain further insights into whether SIN1 mediates AURKA-induced progression of PTC, we conducted western blotting, which showed that the increase in AKT activation caused by AURKA was ablated by knocking down SIN1 (Fig. 6A). AURKA-enhanced malignant behaviours were also repressed by SIN1 depletion, which was confirmed by CCK-8, colony formation experiments, and EdU staining (Fig. 6B–D). To verify whether AURKA exerts biological functions in vivo by regulating SIN1, we constructed a cell line via cotransfection of KTC cells with shSIN1 and AURKA overexpression plasmid. Cells from these three models (KTC overexpression control, KTC overexpression AURKA and KTC overexpression AURKA shSIN1) were subcutaneously injected into nude mice. The growth of subcutaneous tumours was assessed after 2 weeks. The results demonstrated that overexpression of AURKA promoted tumour progression, while SIN1 knockdown attenuated AURKA-elicited malignant phenotype changes in vivo (Fig. 6E–G). The results showed that knockdown of SIN1 could attenuate the promotion of tumour growth by AURKA. Altogether, AURKA exerts a tumour-promoting function by preventing SIN1 from being degraded by the ubiquitin–proteasome system. The malignant biological phenotypes driven by AURKA can be partially restored by decreasing SIN1 expression.

## Discussion

AURKA kinase is a protein kinase associated with centrosome segregation and closely related to cell mitosis; it has been demonstrated to be involved in the development and progression of many solid tumours [23]. Apart from playing a role in mitosis, an increasing number of studies have suggested that AURKA activates many signalling pathways, such as the PI3K/AKT, mTOR, β-catenin/Wnt and NF-κB pathways, and tumorigenesis requires interactions among multiple signalling pathways [9–12]. However, the detailed mechanism of AURKA in PTC progression remains largely unclear. In this study, we described a novel mechanism by which AURKA promotes PTC development by regulating SIN1 protein stability. Importantly, AURKA interacts with SIN1 and counteracts CUL4B-based E3 ligase-mediated degradation. Increased expression of SIN1 promoted the development and progression of PTC by triggering abnormal activation of the mTORC2-AKT pathway (Fig. 6F).

Previous studies have indicated that AURKA is overexpressed in thyroid cancer tissues and cell lines, indicating that it could be helpful for diagnosis and therapy in thyroid cancer [24]. Our results in this study are consistent with previous findings. We found aberrant overexpression of AURKA in PTC, which was highly associated with a poor prognosis and advanced tumour stage, suggesting prognostic value for AURKA. However, one study detected the mRNA level of AURKA in 87 PTC tissues and found that it was not a prognostic biomarker [25]. In our study, we studied the protein level of AURKA using immunohistochemical analysis with a larger cohort including 185 PTC patients. The results showed that patients with high expression of AURKA had poor disease-specific survival. Therefore, multicentre studies should be performed to explore the correlation between the expression of AURKA and clinicopathological features in PTC. In addition, in vitro experiments showed that AURKA promoted malignant behaviours of PTC cells, suggesting an oncogenic role of AURKA. According to in vitro data, AURKA knockdown markedly suppressed thyroid cancer progression in a nude mouse xenograft model. Our results demonstrated that AURKA is essential for the malignancy of PTC. A previous study showed that one AURKA inhibitor, MLN8237, had antitumor effects in thyroid cancer with high c-Myc expression but not in thyroid cancer with low c-Myc expression, suggesting that targeting AURKA may be an effective therapeutic strategy for c-Myc-overexpressing thyroid cancers [18]. Thus, it would be interesting to investigate differences in antitumor mechanisms between different AURKA suppression strategies.

To explore the mechanism of the oncogenic role of AURKA in thyroid cancer cells, we performed whole transcriptome sequencing (RNA-seq) in AURKA knockdown cells and control cells. Notably, differentially expressed genes were significantly enriched in the PI3K/AKT pathway. It is well known that the PI3K/AKT pathway plays vital roles in various cellular processes [26]. Dysfunction of these pathways contributes to many diseases, such as malignant tumours, and some studies have reported that thyroid cancer patients show abnormal activation of PI3K/AKT signalling, which may provide useful therapeutic targets for thyroid cancer [27]. Our results demonstrated that dysregulation of AURKA levels dramatically affected the P-AKT level both in vitro and in vivo. Moreover, the malignant phenotypes induced by AURKA overexpression were partly blocked by AKT inhibitors and mTOR inhibitors, indicating that the oncogenic role of AURKA in thyroid cancer cells partly depends on the PI3K/AKT signalling pathway. Moreover, a number of reports have indicated that AURKA transcription is regulated by MYC downstream of the PI3K/AKT pathway [28]. Therefore, MYC-driven AURKA signalling may constitute a positive feedback loop that helps to continuously activate the PI3K/AKT pathway in thyroid cancer. In addition, it has been reported that AURKA overexpression mediates resistance to PI3K-AKT-mTOR pathway inhibitors in breast cancer. Future work may determine whether AURKA inhibition enhances sensitivity to AKT inhibitors in thyroid cancer.

To further address how AURKA activates the AKT signalling pathway, we then performed IP-MS to identify the proteins that bind to AURKA. Interestingly, our results suggested that AURKA regulates AKT activation by stabilizing the protein level of SIN1, an essential subunit of the mTORC2 complex; maintains the integrity of the complex and substrate specificity; and regulates AKT activation [29]. A previous study revealed that SIN1 was overexpressed in aggressive papillary thyroid cancer and was strongly associated with the activation of AKT kinase. However, the mechanism of SIN1 upregulation in thyroid cancer is not clear. Here, we show that AURKA increases SIN1 protein levels by blocking its degradation via the proteasome pathway. We and others have demonstrated that AURKA promotes cancer progression in a kinase-dependent manner. We also performed phospho-mass spectrometry in AURKA knockdown thyroid cancer cells and control cells. Unfortunately, we did not find that the phosphorylation level of SIN1 was affected after AURKA inhibition (data not shown), indicating that AURKA regulates SIN1 expression in a kinase-independent manner. Our results are in accordance with those of studies showing that AURKA displays a kinase-independent function to promote cancer progression [30]. Further studies are warranted to explore new mechanisms of AURKA in cancer development and develop novel small molecular inhibitors of AURKA.

Another interesting finding is a new ubiquitin E3 ligase of SIN1. Through a mini-library screen of Cullin-RING E3 ligase family members, we observed that CUL4B inhibition obviously increased the protein level of SIN1. CUL4B physically associates with SIN1 and increases its ubiquitination level, thus promoting SIN1 degradation. A previous study showed that the CUL5-SOCS6 E3 complex interacts with SIN1 and regulates its stability in pancreatic cancer cells [31]. In our study, we found that CUL5 inhibition slightly affected SIN1 expression in thyroid cancer cells. One possible explanation for this is the difference in cellular origin and context. CUL4B is a scaffold protein of the CUL4B E3 ligase complex, but the receptor protein that directly interacts with SIN1 has not been identified in our work and requires further investigation.

## Conclusion

In summary, our current work elucidates a crucial role of the AURKA-SIN1-mTORC2/AKT signalling axis in the initiation and progression of PTC. These findings highlight that AURKA is a potential biomarker and a valuable therapeutic target for PTCs.

## Methods

### Cell lines and cell culture

Five of these PTC cell lines (BCPAP, KTC-1, TPC-1, K1 and IHH4) and one normal thyroid follicular epithelial cell line (Nthy-ori 3–1) were used in this study. HEK293T, TPC-1, K1, IHH4 and Nthy-ori 3–1 cells were purchased from the American Type Culture Collection (ATCC, USA), and the other cell lines were purchased from the Type Culture Collection of the Chinese Academy of Sciences (Shanghai, China). The cells in the experiment were all identified by short tandem repeat DNA profiling analysis, and they were all cultured within 20–30 generations, and there was no contamination of Mycoplasma. The cell culture protocol was described in our previous studies.

### Clinical samples

From January 2013 to June 2013, 185 paraffin specimens of patients with PTC confirmed by pathology who underwent surgical treatment at the Cancer Hospital of Tianjin Medical University were selected to make tissue microarrays (TMAs). Clinicopathological data, including sex, age, tumour size, lymph node metastasis, number of tumour lesions, haematological examination, glandular invasion and other clinicopathological characteristics, were collected. TNM staging was based on American Joint Committee on Cancer (AJCC) 8th Edition Differentiated Thyroid Cancer TNM staging. Fresh tissue specimens for real-time qPCR were tumour tissues and matched normal thyroid tissues from 18 PTC patients. The study was conducted under the approval of the Institutional Review Board of Tianjin Medical University Cancer Institute and Hospital.

### Immunohistochemistry staining

TMAs and paraffin sections were stained according to standard immunohistochemical staining methods. The primary antibodies used were anti-AURKA (Abcam, ab1287), anti-Ki67 (CST, #9449), anti-SIN1 (Abcam, ab71152), and anti-P-AKT (CST, #4060). The immunohistochemical final score was the product of the staining intensity and the staining ratio. The intensity was scored as follows: 0, negative; 1, weak; 2, moderate; and 3, strong. The frequency of positive cells was defined as follows: 0, less than 5%; 1, 5% to 25%; 2, 26% to 50%; 3, 51% to 75%; and 4, greater than 75%.

### RNA extraction and quantitative RT–qPCR

TRIzol Reagent (Invitrogen, Carlsbad, CA, USA) was used to extract total RNA from tissues and cell lines, and reverse transcription reagents (TaKaRa, Tokyo, Japan) were used to reverse transcribe the total RNA into cDNA. Specific primers and SYBR Premix Ex Taq II (TaKaRa, Tokyo, Japan) were used to detect the mRNA level of the target gene on the basis of the abovementioned cDNA as a template. The primer sequences are shown in Additional file 2: Table S2.

### Protein extraction and western blot analysis

Proteins from fresh tissues and cell lines were treated with RIPA buffer containing protease inhibitors and phosphatase inhibitors. Protein concentration was determined by the BCA method, and proteins were separated by 8%–12% SDS–PAGE and transferred to polyvinyl difluoride membranes. The membranes were incubated overnight at 4 °C in the corresponding primary antibodies including AURKA (Abcam, ab1287), GAPDH (CST, #5174), SIN1 (CST, #12860), AKT (CST, #4691), P-AKT (CST, #4060), MYC-TAG (CST, #2276), HA-TAG (CST, #2376), Ubiquitin (CST, #3936), Cullin4B (ProteinTech, 12916), HIS-TAG (CST, #12698), DYKDDDDK TAG (CST, #14793). Subsequently, luminescence detection was performed using ECL reagents after incubation with the corresponding secondary antibodies.

### Plasmids and lentiviruses

The lentivirus of shAURKA and Flag-AURKA overexpressing were purchased from Shanghai Genechem company. The lentiviral vector plasmids of MPHV2 and SAMV2 are from the School of Life Sciences, Nankai University. sgRNAs of AURKA were designed and constructed on the plasmid of the SAMV2 lentiviral vector. After the successive infections of MPHv2 and sgRNAs-SAMV2 lentivirus, we obtained a stable integrated cell line of sgRNAs-dcas9-VP64. siRNAs were transfected into cells using Lipofectamine 2000 (Invitrogen, Shanghai, China) according to the manufacturer's instructions. 48 h later, the cells were harvested for further experiments. All the siRNAs were synthesized by Biotend Company, and two independent siRNA duplexes were designed and mixed for transfection to deplete each target gene. Cullin4B shRNA, SIN1 shRNA synthesized by ourselves. All the shRNAs were constructed with pLKO.1 vector. The plasmids for expressing Cullin4B-Myc and Cullin4B-His were generated by inserting the cDNA with Myc tag and His tag into a pCMV vector. The plasmids for expressing SIN1-Myc and SIN1-Myc were generated by inserting the cDNA with Myc tag and His tag into a pCMV vector. Truncated SIN1 as well as Sin1 ubiquitination site mutants were constructed by a pCMV vector. The plasmids for expressing AURKA-Flag were generated by inserting the cDNA with Flag tag into a pCMV vector. The sequences of shRNA, sgRNA, siRNA in Additional file 2: Tables S3–S6.

### Cell viability, colony formation and EdU assays

For the cell survival assay, 1000 cells/well were plated in 96-well plates. Cells were cultivated with media supplemented with 10% FBS. After 24 h, the cells were treated with different concentrations of cisplatin (Selleck, S1166) or gemcitabine (Selleck, S1714) for 24 h. CCK-8 (Meilunbio, MA0218) was then added to the plate and incubated at 37 °C for 2 h. The optical density (OD) was read on a Bio Tek Eon Multi-Mode Microplate Reader. For the colony formation assays, 1000 cells/well were plated in 6-well plates, and the cells were cultivated with media supplemented with 10% FBS. After 10 days, the culture medium was discarded, the cells were fixed with 4% paraformaldehyde for 2 h, the paraformaldehyde was discarded, and the cells were stained with crystal violet for 30 min and then photographed. An appropriate number of cells was seeded in 96-well plates and cultured to the normal growth stage. After incubation with 10 µM EdU solution for 2 h, the cells were fixed with 4% paraformaldehyde. ApolloR staining reaction solution (1×) was incubated for half an hour, and then 1 × Hoechst 33,342 reaction solution (Click-iT EdU Assays, Thermo Fisher, USA) was added. After staining, photos were taken using a fluorescence microscope. All the above operations were performed in the dark.

### Ubiquitylation assay

HEK293T cells were transfected with the indicated plasmids. Mg132 (10 μM) was added to the cells for 12 h. Mg132, a proteasome inhibitor, was purchased from Selleck. Two micrograms of HA-TAG antibody were added to the cell lysates and incubated overnight. The next day, protein A/G was added and incubated for 2 h. After that, the mixture was placed on a magnetic stand, the supernatant was discarded, and the magnetic beads or agarose were washed three times. The samples were added to SDS–PAGE loading buffer and examined by immunoblotting.

### Immunoprecipitation

For immunoprecipitation, 2 μg of antibody was added to cell lysates and incubated overnight. The next day, protein A/G or agarose was added and incubated for 2 h. After that, the mixture was placed on a magnetic stand, the supernatant was discarded, and the magnetic beads or agarose were washed three times. Loading buffer was added to the tube and heated at 100 °C for 15 min prior to immunoblot analysis.

### Tumour xenografts

Six- to eight-week-old male BALB/c nude mice were purchased from Beijing Vital River Laboratory Animal Technology Company (Vital River, Beijing, China) and were acclimated for 7 days in the animal facility before experiments. A total of 1 × 10^6^ TPC-1 cells stably transfected with shAURKA or control PLKO and 1 × 10^7^ KTC cells stably transfected with OE AURKA or control PCDH were injected subcutaneously into the dorsal flanks of the mice (n = 5). Tumour volume was measured approximately every 3 days and calculated according to the following formula: Volume (mm^3^) = 1/2 × length × width^2^. Mice were sacrificed, and tumour tissues were collected once the tumour length reached 10 mm. Haematoxylin–eosin staining and immunohistochemistry were performed on processed and sectioned tissues. All procedures were approved by the Animal Care Committee of Tianjin Medical University Cancer Hospital.

### RNA-seq analysis and immunoprecipitation-mass spectrometry

After transfection with shAURKA, TPC-1 cells were lysed using TRIzol reagent (Invitrogen, Carlsbad, CA, USA). The global gene expression profiles were detected by mRNA sequencing on the Illumina HiSeq Xten sequencing platform (Majorbio Biopharm Technology Co., Ltd, Shanghai, China). Bioinformatics analyses were implemented as described previously [22]. IP-purified protein complexes were separated by SDS–PAGE. Differential bands were cut and analysed by LC–MS at PTM BIO. MS data analysis to select potential candidate validation proteins.

### Statistical analysis

All data are expressed as the mean ± SD from three independent experiments, unless specified, and each experiment was performed at least in triplicate. Statistical significance was evaluated with a two-tailed paired t test by using SPSS 17.0 software. A p value of < 0.05 was considered to indicate significance.

## Supplementary Information

**Additional file 1**: **Figure S1.** Expression level of AURKA in thyroid cancer cell lines. (A) Quantitative real-time PCR to measure the mRNA level of AURKA in normal thyroid cells and thyroid cancer cells. (B) The protein expression levels of AURKA were examined by western blotting in normal thyroid cells and thyroid cancer cells. **Figure S2.** AURKA plays oncoprotein roles in PTC. (A)Schematic of CRISPR Cas9 technology. (B) The protein level of AURKA was detected in AURKA knockdown IHH4 cells and AURKA-overexpressing K1 cells by western blotting.(C) The viability of shAURKA-transfected IHH4 cells or AURKA-overexpressing K1 cells compared with control cells was detected using the CCK-8 assay. (D) The ratio of EdU-labelled cells to total cells was observed in AURKA knockdown IHH4 cells and AURKA-overexpressing K1 cells compared with the control group (original magnification x20). (E) shAURKA-transfected IHH4 cells and AURKA overexpressing K1 cells were cultured for 10 days prior to crystal violet staining. (F) Transwell migration assays were used to detect the migration ability of AURKA knockdown IHH4 cells and AURKA-overexpressing K1 cells compared with control cells. (G) The expression levels of AURKA, Ki67 in xenografts of each group were assessed by immunohistochemical staining. The scale bar is 50 μm. Data are shown as the mean ± SD of three replicates (*P < 0.05, **P < 0.01, ***P < 0.001). **Figure S3.** AURKA affects the protein stability of SIN1. (A)Western blotting validation the P13K/AKT signaling pathway in AURKA knockdown IHH4 cells and AURKA-overexpressing K1 cells. (B) The expression levels of P-AKT in xenografts of each group were assessed by immunohistochemical staining. The scale bar is 50 μm. (C) In TPC1 cells, AURKA (green) was immunofluorescent with SIN1 (red), and AURKA was found to colocalize with SIN1 (yellow). (D) The protein expression levels of SIN1 were examined by western blotting in shAURKA-transfected IHH4 cells and AURKA-overexpressing K1 cells. (E) The expression levels of SIN1 in xenografts of each group were assessed by immunohistochemical staining. The scale bar is 50 μm. (F) The RNA expression levels of SIN1 were examined by quantitative real-time PCR in AURKA-overexpression plasmid-transfected K1 cells or KTC cells and shAURKA-transfected TPC-1 cells and IHH4 cells. **Figure S4.** CUL4B affects the protein stability of SIN1. (A) The RNA expression levels of Cullins were examined by quantitative real-time PCR in siRNA-transfected TPC-1 cells. (B) The protein expression of SIN1 in TPC-1 cells treated with 200 μM CHX for different durations was measured by western blotting analysis. (C) 293T cells were transfected with the indicated plasmids and siRNA, and the protein expression was examined by western blotting. MG132 (20 μM) was added 6 hrs prior to harvest.

**Additional file 2**: **Table S1**, **Table S2.** qRT-PCR primer sequences in this study. **Table S3.** sgRNA sequences in this study. **Table S4.** siRNA sequences in this study. **Table S5.** shRNA sequences in this study. **Table S6.** Primer sequences in this study

## Data Availability

All data generated or analysed during this study are included in this published article and its supplementary information files.

## Abbreviations

AURKA: Aurora kinase A
mTOR: Mechanistic target of rapamycin
mTORC2: Mechanistic target of rapamycin complex 2
AKT: Protein Kinase B
IKK: Inhibitor of kappa B kinase
NF-ΚB: Nuclear factor kappa-B
PI3K: Phosphatidylinositol3-kinase
MMP: Mitochondrial membrane potential
MAPK: MAP kinase kinase kinase
SIN1: Stress-activated protein kinase (SAPK) interacting protein 1
PTC: Papillary thyroid carcinoma
TC: Thyroid carcinoma
