# Supplementary material for: Aurora kinase a promotes the progression of papillary thyroid carcinoma by activating the mTORC2-AKT signalling pathway
Source: Cell Biosci. 2022 Dec 5;12:195. doi: 10.1186/s13578-022-00934-z (PMC9721059; doi:10.1186/s13578-022-00934-z)
Supplement: Supplementary file 1 — Additional file 1: Figure S1. Expression level of AURKA in thyroid cancer cell lines. (A) Quantitative real-time PCR to measure the mRNA level of AURKA in normal thyroid cells and thyroid cancer cells. (B) The protein expression levels of AURKA were examined by western blotting in normal thyroid cells and thyroid cancer cells. Figure S2. AURKA plays oncoprotein roles in PTC. (A)Schematic of CRISPR Cas9 technology. (B) The protein level of AURKA was detected in AURKA knockdown IHH4 cells and AURKA-overexpressing K1 cells by western blotting.(C) The viability of shAURKA-transfected IHH4 cells or AURKA-overexpressing K1 cells compared with control cells was detected using the CCK-8 assay. (D) The ratio of EdU-labelled cells to total cells was observed in AURKA knockdown IHH4 cells and AURKA-overexpressing K1 cells compared with the control group (original magnification x20). (E) shAURKA-transfected IHH4 cells and AURKA overexpressing K1 cells were cultured for 10 days prior to crystal violet staining. (F) Transwell migration assays were used to detect the migration ability of AURKA knockdown IHH4 cells and AURKA-overexpressing K1 cells compared with control cells. (G) The expression levels of AURKA, Ki67 in xenografts of each group were assessed by immunohistochemical staining. The scale bar is 50 μm. Data are shown as the mean ± SD of three replicates (*P < 0.05, **P < 0.01, ***P < 0.001). Figure S3. AURKA affects the protein stability of SIN1. (A)Western blotting validation the P13K/AKT signaling pathway in AURKA knockdown IHH4 cells and AURKA-overexpressing K1 cells. (B) The expression levels of P-AKT in xenografts of each group were assessed by immunohistochemical staining. The scale bar is 50 μm. (C) In TPC1 cells, AURKA (green) was immunofluorescent with SIN1 (red), and AURKA was found to colocalize with SIN1 (yellow). (D) The protein expression levels of SIN1 were examined by western blotting in shAURKA-transfected IHH4 cells and AURKA-overexpressing K1 cells. ( [file 13578_2022_934_MOESM1_ESM.doc]

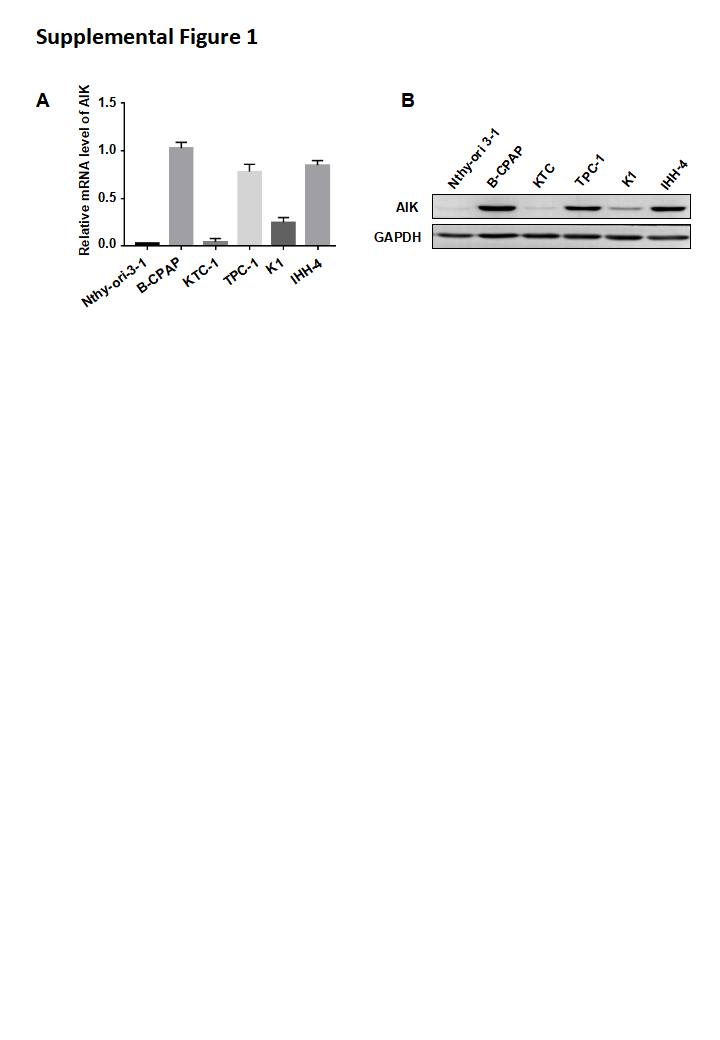


**Supplemental Figure 1. Expression level of AURKA in thyroid cancer cell lines.** **(A)** Quantitative real-time PCR to measure the mRNA level of AURKA in normal thyroid cells and thyroid cancer cells. **(B)** The protein expression levels of AURKA were examined by western blotting in normal thyroid cells and thyroid cancer cells.


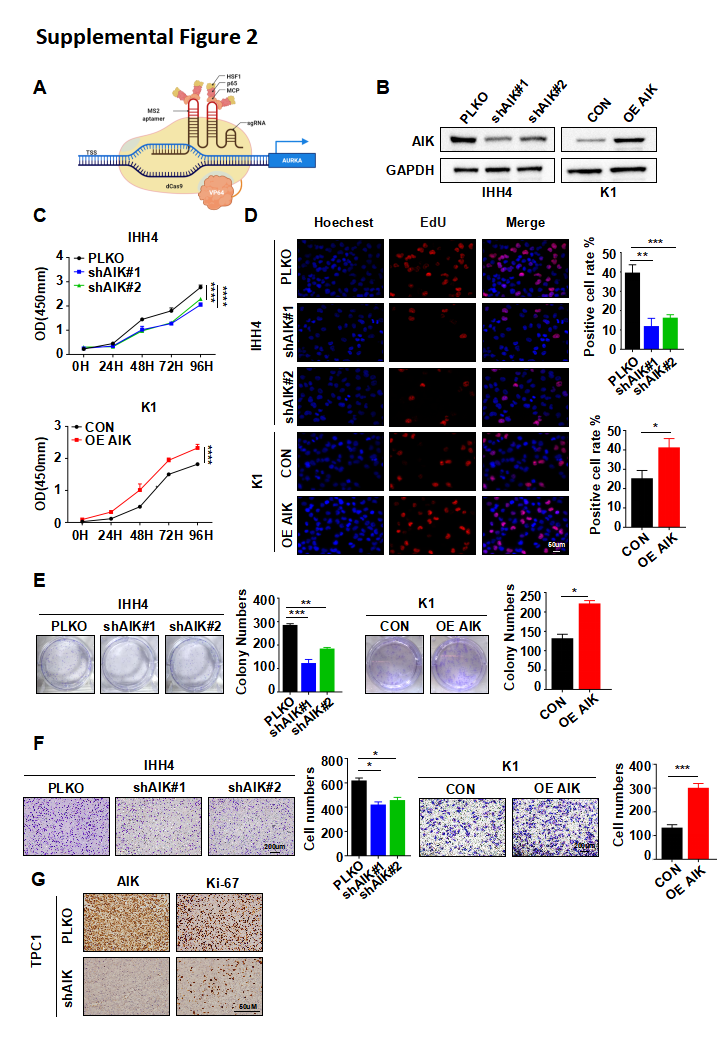


**Supplemental Figure 2. AURKA plays oncoprotein roles in PTC.** **(A)**Schematic of CRISPR Cas9 technology. **(B)** The protein level of AURKA was detected in AURKA knockdown IHH4 cells and AURKA-overexpressing K1 cells by western blotting.**(C)** The viability of shAURKA-transfected IHH4 cells or AURKA-overexpressing K1 cells compared with control cells was detected using the CCK-8 assay. **(D)** The ratio of EdU-labelled cells to total cells was observed in AURKA knockdown IHH4 cells and AURKA-overexpressing K1 cells compared with the control group (original magnification x20). **(E)** shAURKA-transfected IHH4 cells and AURKA overexpressing K1 cells were cultured for 10 days prior to crystal violet staining. **(F)** Transwell migration assays were used to detect the migration ability of AURKA knockdown IHH4 cells and AURKA-overexpressing K1 cells compared with control cells. **(G)** The expression levels of AURKA, Ki67 in xenografts of each group were assessed by immunohistochemical staining. The scale bar is 50 μm. Data are shown as the mean ± SD of three replicates (*P < 0.05, **P < 0.01, ***P < 0.001).

**
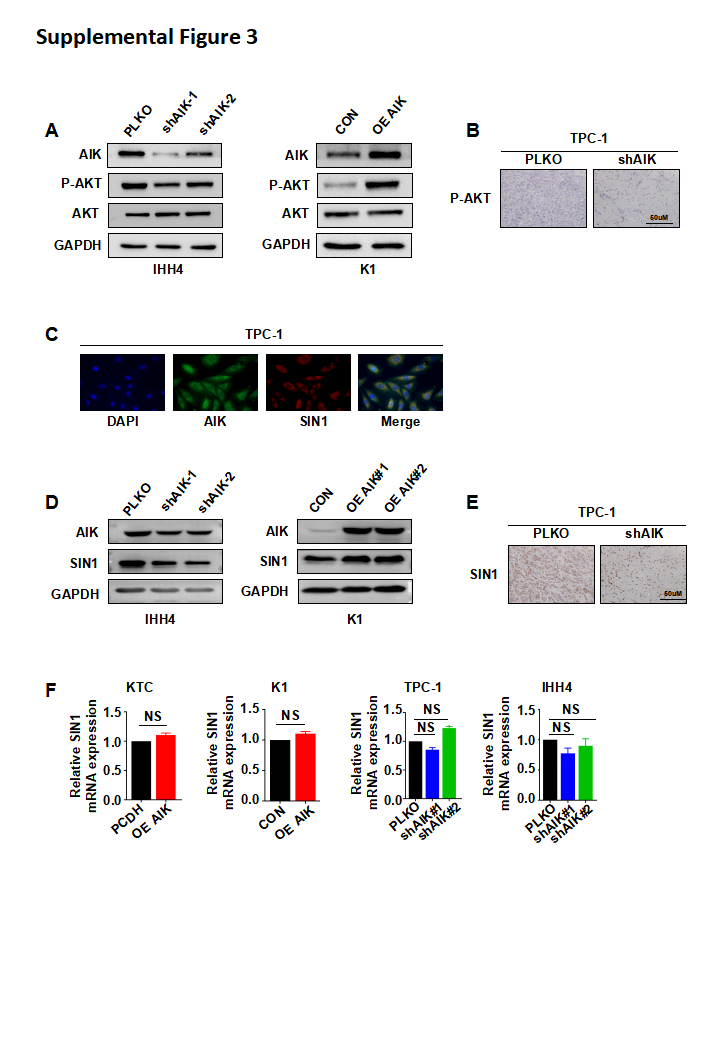
**

**Supplemental Figure 3. AURKA affects the protein stability of SIN1. (A)**Western blotting validation the P13K/AKT signaling pathway in AURKA knockdown IHH4 cells and AURKA-overexpressing K1 cells. **(B)** The expression levels of P-AKT in xenografts of each group were assessed by immunohistochemical staining. The scale bar is 50 μm. **(C**) In TPC1 cells, AURKA (green) was immunofluorescent with SIN1 (red), and AURKA was found to colocalize with SIN1 (yellow). **(D)** The protein expression levels of SIN1 were examined by western blotting in shAURKA-transfected IHH4 cells and AURKA-overexpressing K1 cells. **(E)** The expression levels of SIN1 in xenografts of each group were assessed by immunohistochemical staining. The scale bar is 50 μm. **(F)** The RNA expression levels of SIN1 were examined by quantitative real-time PCR in AURKA-overexpression plasmid-transfected K1 cells or KTC cells and shAURKA-transfected TPC-1 cells and IHH4 cells.

**
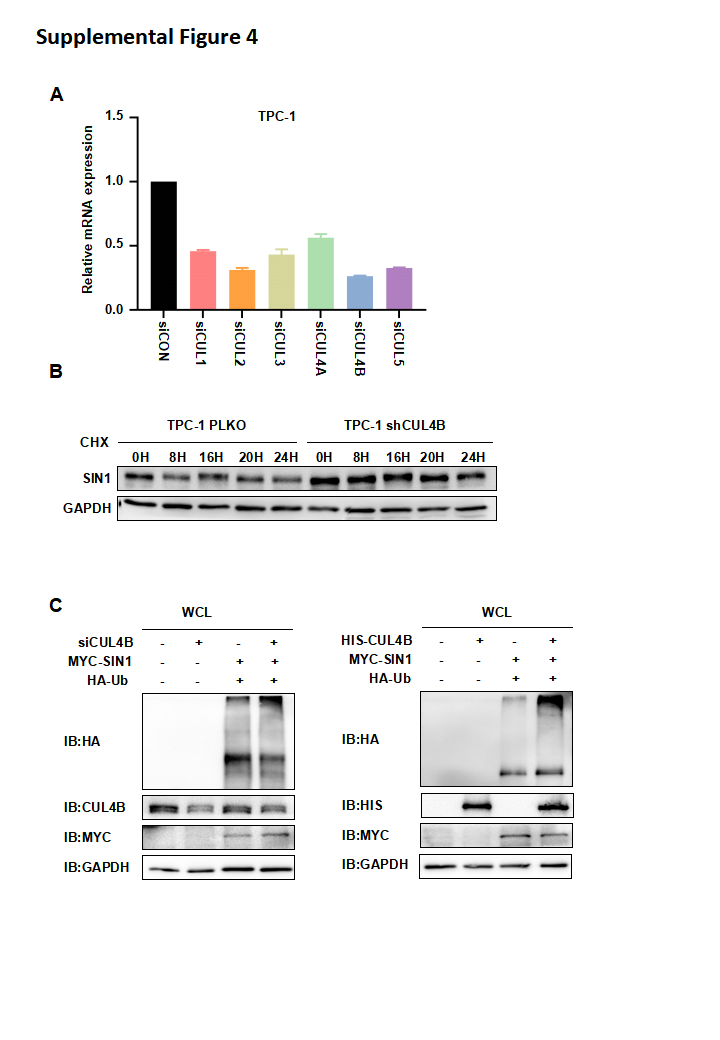
**

**Supplemental Figure 4. CUL4B affects the protein stability of SIN1. (A)** The RNA expression levels of Cullins were examined by quantitative real-time PCR in siRNA-transfected TPC-1 cells. **(B)** The protein expression of SIN1 in TPC-1 cells treated with 200 μM CHX for different durations was measured by western blotting analysis. **(C)** 293T cells were transfected with the indicated plasmids and siRNA, and the protein expression was examined by western blotting. MG132 (20 μM) was added 6 hrs prior to harvest.
