# Supplementary material for: Aurora kinase a promotes the progression of papillary thyroid carcinoma by activating the mTORC2-AKT signalling pathway
Source: Cell Biosci. 2022 Dec 5;12:195. doi: 10.1186/s13578-022-00934-z (PMC9721059; doi:10.1186/s13578-022-00934-z)
Supplement: Supplementary file 2 — Additional file 2: Table S1, Table S2. qRT-PCR primer sequences in this study. Table S3. sgRNA sequences in this study. Table S4. siRNA sequences in this study. Table S5. shRNA sequences in this study. Table S6. Primer sequences in this study [file 13578_2022_934_MOESM2_ESM.doc]

**Supplementary Table 1:**

**Variables Low expression High expression χ^2^ P**

**Age**

**＜55 60 9 14.666 ＜0.001**

**＞=55 70 46**

**Sex**

**Female 89 43 1.786 0.181**

**Male 41 12**

**Multifocality**

**Presrent 41 17 0.007 0.933**

**Absent 89 38**

**Tumor stage**

**T_1/2_ 121 46 3.922 0.048**

**T_3/4_ 9 9**

**Lymph node metastasis**

**N_0_ 74 15 13.611 ＜0.001**

**N1_a/b_ 56 40**

**AJCC stage**

**I+II 126 46 10.443 0.001**

**III+IV 4 9**

**Recurrence**

**Absent 116 39 9.549 0.002**

**Present 14 16**

**Supplementary Table 2: qRT-PCR primer sequences in this study**

**Primer names Primer sequence**

**GAPDH-F GGAGCGAGATCCCTCCAAAAT**

**GAPDH-R GGCTGTTGTCATACTTCTCATGG**

**Aurora-A-F GAGGTCCAAAACGTGTTCTCG**

**Aurora-A-R ACAGGATGAGGTACACTGGTTG**

**SIN1-F AAGCAATCAGCCCAGGAGTT**

**SIN1-R TATTCAGCTGCAGAGGGCAC**

**CUL1-F CAATGACGCTGGCTTTGTGGCT**

**CUL1-R CAAGGAGTCACAGTATCGAGCC**

**CUL2-F GTCTTACTCCGTGCTGTGTCCA**

**CUL2-R CTGACTCCACAAATAGTGTTGGC**

**CUL3-F TCGACAGCTCACACTCCAGCAT**

**CUL3-R GTGCTTCCGTGTATTAGAGCCAG**

**CUL4A-F GAATGAGCGGTTCGTCAACCTG**

**CUL4A-R CTGTGGCTTCTTTGTTGCCTGC**

**CUL4B-F GAAGCTACAGATGAAGAACTTGAG**

**CUL4B-R GCACTCTTTCCGACTAACAGGC**

**CUL5-F CCTGATGCTGAACTTAGGAGGAC**

**CUL5-R GGTTCACTGAGAAGAGGGTACC**

**Supplementary Table 3: sgRNA sequences in this study**

**Primer names Primer sequence**

**Aurora-a1-sg-F CACCGCGACCGCGGAGAGCACCCTG**

**Aurora-a1-sg-R AAACCAGGGTGCTCTCCGCGGTCGC**

**Aurora-a2-sg-F CACCGGCGCCCTTAAACGCGACTCA**

**Aurora-a2-sg-R AAACTGAGTCGCGTTTAAGGGCGCC**

**Supplementary Table 4: siRNA sequences in this study**

**Primer names Primer sequence**

**siCUL1-F GGAUGAGAGUGUACUGAAA**

**siCUL1-R CCAGAUAUAUGGAGCUCUA**

**siCUL2-F GGCAAAUAUGUACGUCUUA**

**siCUL2-R GAAGAACAAGUACUUGUUA**

**siCUL3-F AAGGUGCGAGAAGAUGUATT**

**siCUL3-R AACAACUUUCUUCAAACGCUA**

**siCUL4A-F GGAAGAGACUAAUUGCUUA**

**siCUL4A-R GAACAGCGAUCGUAAUCAA**

**siCUL4B CCACCCAGAAGUCAUUAAUTT**

**siCUL4B-F GGCUAAUAGAGCACAAAUA**

**siCUL4B-R CCAGCUGAUUCAGUUAAUA**

**Supplementary Table 5: shRNA sequences in this study**

**Primer names Primer sequence**

**shSIN1-F GCACACAAGATGAATTAGCAA**

**shSIN1-R TTGCTAATTCATCTTGTGTGC**

**shCullin4B-F GCTGAATTTAAAGAGGGCAAA**

**shCullin4B-R TTTGCCCTCTTTAAATTCAGC**

**shAIK-1-F CCTGTCTTACTGTCATTCGAA**

**shAIK-1-R TTCGAATGACAGTAAGACAGG**

**shAIK-2-F GAGTCTACCTAATTCTGGAAT**

**shAIK-2-R ATTCCAGAATTAGGTAGACTC**

**Supplementary Table 6: Primer sequences in this study**

**Primer names Primer sequence**

**PCDH-SIN1-F GGAATTCCATGGCCTTCTTGGACAATCCAAC**

**PCDH-SIN1-R TTGCGGCCGCAACAGATCCTCTTCAGAGATGA GTTTCTGCTCTCACTGCTGCCCGGATTT**

**PCDH-SIN1-N-F GGAATTCCATGGAGCAGAAACTCATCTCTGAA GAGGATCTGATGAAAGAGAAGCCTCCA**

**PCDH-SIN1-CRIM-R ATGAGTACTTAGAAATTGGAGGCTTCT**

**PCDH-SIN1-CRIM-F TCCAATTTCTAAGTACTCATCTCCTGG**

**PCDH-SIN1-RBD-R AACTGTTCTCTGACTCTTTGGATGTCA**

**PCDH-SIN1-RBD-F CAAAGAGTCAGAGAACAGTTCAAGGGC**

**PCDH-SIN1-PH-R AACTGTTCTCGCGGACCAG**

**PCDH-SIN1-PH-F CCTGGTCCGCGAGAACAGTT**

**PCDH-CUL4B-F CGGGATCCCGATGCATCATCACCATCACCATA TGTTTCCAACAGG**

**PCDH-CUL4B-R TTGCGGCCGCAACTATGCAATATAGTTGTACT GGTTTGGATTTTCTT**

**PCDH-SIN1-121-F CAGCCCAGGAGTTAAGATCACTGTTTGAAAAA**

**PCDH-SIN1-121-R TTTTTCAAACAGTGATCTTAACTCCTGGGCTG**

**PCDH-SIN1-162-F TTAACGAGTATTCCAGATTTGATGGCAAGGG**

**PCDH-SIN1-162-R CCCTTGCCATCAAATCTGGAATACTCGTTAA**

**PCDH-SIN1-166-F CCAAATTTGATGGCAGAGGTCATGTAGGTAC**

**PCDH-SIN1-166-R GTACCTACATGACCTCTGCCATCAAATTTGG**

**PCDH-SIN1-276-F CTGGTCTGACATCCAGAGAGTCACTCTT**

**PCDH-SIN1-276-R AAGAGTGACTCTCTGGATGTCAGACCAG**

**PCDH-SIN1-302-F CAAAGGTTACCATGAGAGAAATCTTACTGAA**

**PCDH-SIN1-302-R TTCAGTAAGATTTCTCTCATGGTAACCTTTG**

**PCDH-SIN1-428-F TTGGATTAAGCAGAGACCCATCTCAATCG**

**PCDH-SIN1-428-R CGATTGAGATGGGTCTCTGCTTAATCCAA**

**PCDH-SIN1-501-F ACTACTTTGCTCAAAGACAAAGAAAACTGAA**

**PCDH-SIN1-501-R TTCAGTTTTCTTTGTCTTTGAGCAAAGTAGT**
